# Assessment of the Impact of Physical Activity on the Musculoskeletal System in Early Degenerative Knee Joint Lesions in an Animal Model

**DOI:** 10.3390/ijms24043540

**Published:** 2023-02-10

**Authors:** Jaromir Jarecki, Izabela Polkowska, Waldemar Kazimierczak, Magdalena Wójciak, Ireneusz Sowa, Sławomir Dresler, Tomasz Blicharski

**Affiliations:** 1 Department of Rehabilitation and Orthopaedics, Medical University of Lublin, 20-059 Lublin, Poland; 2 Department and Clinic of Animal Surgery, University of Life Sciences, 20-033 Lublin, Poland; 3 Department of Biomedicine and Environmental Research, Faculty of Medicine John Paul II, Catholic University of Lublin, 20-708 Lublin, Poland; 4 Department of Analytical Chemistry, Medical University of Lublin, Aleje Raclawickie 1, 20-059 Lublin, Poland; 5 Department of Plant Physiology and Biophysics, Institute of Biological Sciences, Maria Curie-Skłodowska University, 20-033 Lublin, Poland

**Keywords:** sarcopenia, osteoarthritis, musculoskeletal system

## Abstract

Osteoarthritis (OA) is one of the most prevalent diseases of the osteoarticular system. Progressive destruction of joints is accompanied by development of pathological changes in the muscle tissue, i.e., weakening, atrophy, and remodelling (sarcopenia). The aim of the present study is to assess the impact of physical activity on the musculoskeletal system in an animal model of early degenerative lesions in the knee joint. The study involved 30 male Wistar rats. The animals were allocated to three subgroups of 10 animals each. Each animal from the three subgroups received sodium iodoacetate by injection into the patellar ligament of the right knee joint, whereas saline was administered through the patellar ligament in the left knee joint. The rats in the first group were stimulated to exercise on a treadmill. The animals in the second group were allowed to lead a natural lifestyle (no treadmill stimulation). In the third group, all parts of the right hind limb muscle were injected with Clostridium botulinum toxin type A. The study demonstrated that, compared to the active rats, bone density in the immobilised rats decreased, as indicated by the densitometric assessment of the whole body and the examination of rats’ hind limbs and knee joints alone. This clearly evidenced the impact of physical activity on bone mineralisation. The weight of both fat and muscle tissues in the physically inactive rats was reduced. Additionally, the adipose tissue had higher weight in the entire right hind limbs, where monoiodoacetic acid was administered to the knee joint. The animal model clearly showed the importance of physical activity in the early stages of OA, as it slows down the process of joint destruction, bone atrophy, and muscle wasting, whereas physical inactivity contributes to progression of generalised changes in the musculoskeletal system.

## 1. Introduction

Osteoarthritis (OA) is one of the most prevalent inflammatory diseases of the osteoarticular system and one of the leading causes of disability worldwide, affecting approximately 250 million people [1,2,3]. It causes joint pain, limits physical and social activity, and often leads to chronic disability [2]. 

Current concepts define OA as a whole joint disease with a multifactorial pathogenesis involving biochemical, morphological, molecular, and biomechanical changes in both the extracellular matrix (ECM) and cartilage cells, inflammation of the synovial membrane, sclerotic changes in subchondral bone, meniscal and ligament damage, formation of bone osteophytes, Hoffa fat pad inflammation, and developing fibrosis [4,5,6,7,8].

Bone tissue remodelling processes are carried out by bone cells. Osteoblasts are involved in bone formation and repair, osteoclasts, i.e., phagocytic cells, are responsible for bone destruction, and osteocytes serve a mechanosensitive function (by sensing bone tissue tension during muscle work) [9]. Interactions between these cells facilitate the maintenance of proper metabolism and bone tissue remodelling. Their activity may be influenced by various extrinsic and intrinsic factors. One of the external factors is physical activity. In many studies, authors emphasise that inactivity or reduced physical activity decrease muscle function and contractility. This is reflected by changes in the hormonal system. The absence of exercise leads to reduced concentrations of testosterone, oestrogen, or growth hormone [10,11], and increased concentrations of pro-inflammatory cytokines, such as interleukin-1 (IL-1), IL6, and tumour necrosis factor alpha (TNF-α) [12]. These processes lead to reduction in the activity of osteoblasts and activation of osteoclasts, which may initiate changes resulting in a secondary decrease in bone tissue density, disturbances in bone mineralisation, and development of osteoporosis [13]. 

There are many available options for OA treatment and alleviation of symptoms. The application of non-steroidal anti-inflammatory drugs (NSAIDs), analgesics, or intra-articular steroid injections brings short-term therapeutic effects. Novel methods for treatment of joint cartilage with chondroitin and glucosamine sulphates, hyaluronic acids, platelet-rich preparations (PRP), or mesenchymal stem cells (MSC) improve the status of cartilage tissue and may slow down and alleviate the disease process [14]. The progression of the disease and reduction in pain may be influenced by rehabilitation, physical activity, exercise, weight loss, and modification of the lifestyle. However, none of these measures have the potential to regenerate articular cartilage [15,16]. 

The musculoskeletal system is a diverse group of tissues, including muscles, bones, tendons, ligaments, and cartilage, which work together to maintain physical function. Similar to the aging process, inactivity or limitation of physical activity may induce natural thinning of bone tissue, which results in the development of osteoporosis. Additionally, the structure and hydration of articular cartilage may change, which leads to development of degenerative joint lesions [17]. Ligaments and tendons surrounding joints stiffen and thus limit their mobility and slow down their movement. Moreover, contraction, atrophy, and fatty degeneration of muscle fibres may occur, which reduces muscle tone and strength and leads to the development of secondary sarcopenia [18].

Regular physical activity maintains the proper function of the musculoskeletal system. It has an impact on cartilage nutrition, maintenance or growth of muscle mass, and remodelling and quality of bone tissue [19,20]. Numerous studies have shown a relationship between the sedentary lifestyle, obesity, and chronic inflammation and their negative impact on the progression and severity of OA. It has been proved that the sedentary lifestyle enhances the severity of arthralgia [21]. Muscle strength and muscle mass tend to decline without regular physical activity. The peak muscle mass is reached around the age of 30. It is followed by a regular 3–8% decrease per decade, with an acceleration of the process after the age of 60. Sarcopenia was defined in 1989 as the progressive loss of muscle mass with advancing age [22]; however, it was shown to be an inadequate description of the syndrome [23], as this definition did not take muscle function into account.

The most widely cited definition nowadays is that proposed by the European Working Group on Sarcopenia in Older People (EWGSOP), which was updated in January 2019. This is the only definition endorsed by a range of international scientific societies for clinical practice and research [24]. In clinical practice, the EWGSOP2 states that a person with low muscle strength and low muscle mass or quality will be diagnosed with sarcopenia. The condition can be best understood as skeletal muscle failure or insufficiency [25].

Sarcopenia affects at least 20% of the population over the age of 70 and more than 50% of those over the age of 75. The condition mainly affects the hind limbs [26].

A high probability of development and progression of sarcopenia has been observed in patients with OA, which may be related to changes in the concentration of pro-inflammatory cytokines [27]. An increase in the concentration of IL-1 beta or TNF alpha accelerates protein catabolism, which may explain the loss of muscle mass associated with the development of OA [27,28]. A combination of degenerative lesions in joints with progressive sarcopenia ultimately contributes to loss of independence and, consequently, to a higher degree of dependence on others [26]. Moderate physical exercise stimulates chondrocytes to produce extracellular matrix substances and contributes to normal development and metabolism of cartilage. In turn, excessive physical activity may lead to activation of apoptotic processes and destruction of mechanosensitive cartilage cells [29,30].

Given its ability to assess the body mass ratio accurately, dual-energy X-ray absorptiometry (DXA) is currently the most commonly used tool for evaluation of sarcopenia [28,31]. Although many studies have been carried out [26,32,33,34,35] the relationship between OA and sarcopenia is still unclear. In 2014, Papalia et al. [26] claimed that neither the thesis of a direct effect of sarcopenia on OA development nor the opposite relation can be supported, because the up-to-date literature lacks basic science studies of these topics. Lee [32] observed that a low skeletal muscle mass index in the legs is an independent risk factor for knee OA. Toda [33] indicated that older women with knee OA have a markedly lower percentage of lean body mass compared with their peers without knee OA. Veronese et al. [34] suggested that sarcopenia could be associated with a higher risk of negative knee OA outcomes and, in particular, symptomatic forms. Peng et al. [35] confirmed the relationship between the occurrence of OA and sarcopenia, which nevertheless requires further research, as suggested by the authors.

The aim of the present study is to assess the impact of physical activity on the musculoskeletal system in a rat model of early degenerative lesions in the knee joint induced with monoiodoacetic acid. 

## 2. Results

### 2.1. Radiological Examination

**Figure 1:**
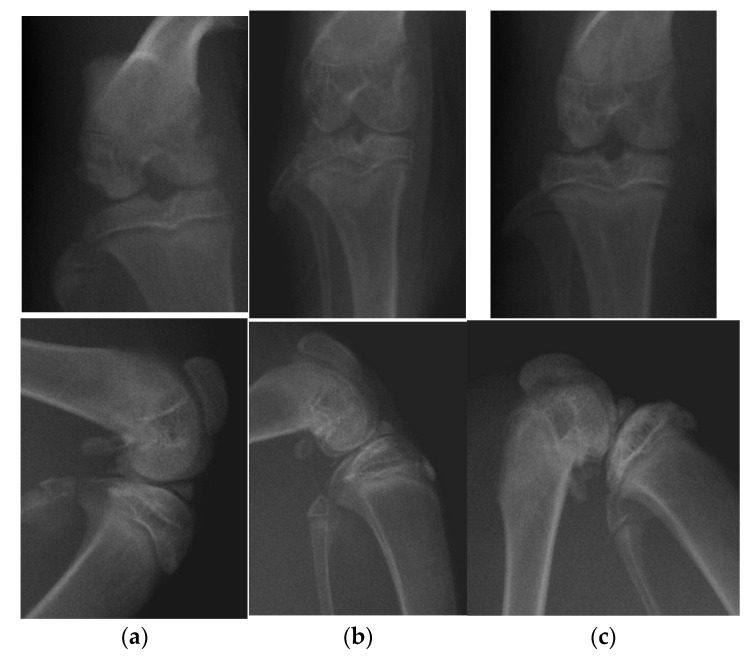
Antero-posterior (**upper panel**) and lateral (**lower panel**) radiographs of the right knee joint of (**a**) running rat, (**b**) immobilised rat, (**c**) walking rat.

X-ray images were used to observe osteoarthritis-like lesions induced by monoiodoacetic acid (MIA) in each group (Figure 1). 

There were no differences in the progression of the degenerative changes shown by the X-ray images in all the examined animal groups, and there were no distinct osteophytic lesions at the edges of the femoral, tibial, and patellar bones. Based on Kellgren–Lawrence classification [36,37], all objects were classified as grade 1. 

### 2.2. Impact of Physical Activity on the Musculoskeletal System—The Whole Body Parameters

**Figure 2:**
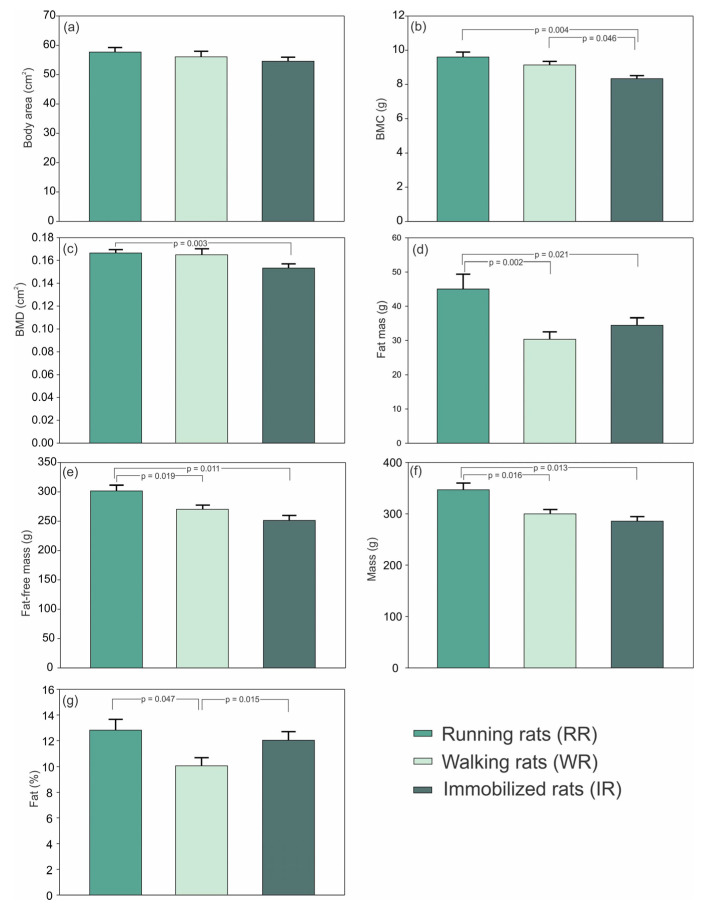
Mean (±SD) parameters of the whole body of rats treated with different types of movements. (**a**) body area (cm^3^); (**b**) BMC—bone mineral content (g); (**c**) BMD—bone mineral density (cm^2^); (**d**) fat mass (g); (**e**) fat-free mass (g); (**f**) mass (g); (**g**) fat (%). Ten animals were included in each group.

The results for all investigated groups are shown in Figure 2.

In the study, no significant relationship was observed between the physical activity and the body area parameter (Figure 2a). The results showed that the immobilisation of the rats decreased the BMC value in comparison with RR and WR: RR vs. IR *p* = 0.0043; WR vs. IR *p* = 0.0458; RR vs. WR *p* = 0.3023 (Figure 2b) and the BMD value in comparison with RR only: RR vs. IR *p* = 0.0026 (Figure 2c). A significant increase in the body weight was observed in the running rats (RR) in comparison with WR and IR: RR vs. WR *p* = 0.0164, RR vs. IR *p* = 0.0132 (Figure 2f). The rats from the RR group exhibited a significantly higher value of fat mass: RR vs. WR *p* = 0.0471, WR vs. IR *p* = 0.0153 (Figure 2g) and free fat mass compared with the WR and IR groups: RR vs. WR *p* = 0.0017, RR vs. IR *p* = 0.0.0211 (Figure 2d), RR vs. WR *p* = 0.0189, RR vs. IR *p* = 0.0107 (Figure 2e).

### 2.3. Impact of Physical Activity on the Musculoskeletal System—Limb and Knee Parameters

The comparison of the results obtained for the hind limbs and knee joints with MIA induced OA (right side) and for control (left side) are shown in Table 1 and Table 2, respectively.

The comparison of both the right and left hind limbs revealed statistically significant differences in the BMC, BMD, fat-free mass, and total mass parameters only in the immobilised group (IR) (Table 1). However, this may be associated with the intramuscular application of the botulinum toxin, as there were no significant differences in the investigated parameters (Table 1) in the joints between the right and left sides for RR and WR. 

The comparison of both the right and left knee joints demonstrated statistically significant differences in the area, BMC, BMD, fat-free mass, and fat% concentration in the immobilised rats (IR). It was also found that the values of the fat mass and the fat% concentration parameters were statistically significant in the group of walking rats (WR).

The comparison of the results between the investigated groups of animals is presented in Table 3.

A difference was found in the knee joint area between the RR and IR groups. A decrease in BMC in both the limbs and the knee joints was observed in the group of the immobilised rats compared with the RR and WR groups. The BMD value in the hind limb and knee joint was found to decrease in the IR group in comparison with the RR and WR groups. There was a difference in the knee joint fat mass between the WR and RR groups and a difference in the fat mass of the hind limb and knee joint between the WR and IR groups. Additionally, there were differences in the fat-free mass in the knee joints between the groups of running and immobilised rats. An increase in the knee joint mass was observed in the group of running rats compared with the immobilised animal group. Differences were found in the limb fat% concentrations between the WR and IR groups and in the knee joint fat% between the RR and IR groups.

### 2.4. Principle Component Analysis (PCA)

**Figure 3:**
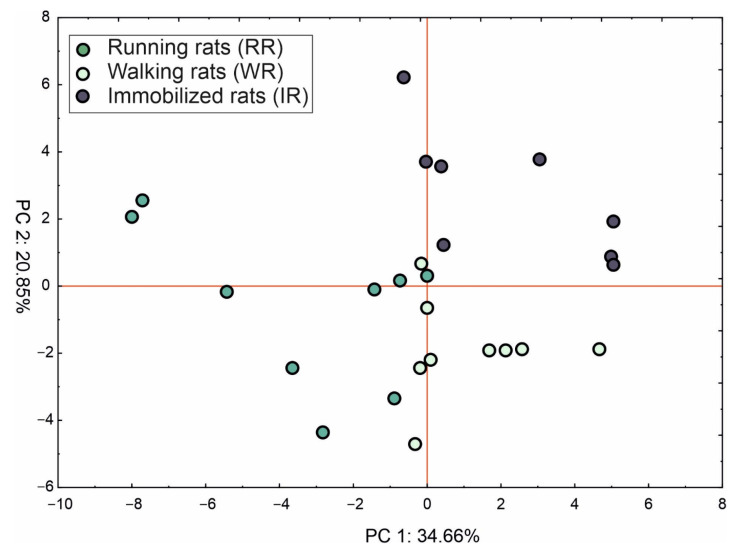
Principal component analysis of whole body parameters in the rats and the variables of both the right and left knee and the limb.

Figure 3 displays the PCA score plots, while Table S1 shows loading of the parameters of the whole body and both the knee and limb results. The first two principle components are responsible for 55.5% of the total variance. The first component explained 34.7% of total variance and was negatively correlated with all estimated variables. Generally, PC1 facilitated separation of the RR individuals (left side of the PC1 axis) from both WR and IR individuals. In turn, the second component (20.9% of total variance) separated the WR group (individuals with hind limb fat mass and percent of fat in both the right and left limb and knee joint) from the IR individuals (lower BMD and BMC of the right knee joint and limb and a lower sum of fat-free and total mass of the knee joint).

## 3. Discussion

There are many methods for induction of degenerative lesions in the joints. One of the most common substances used in animals is monoiodoacetic acid [38]. MIA injected into the joint causes chondrocyte death and changes in bone tissue structure and in the subchondral layer [39]. Inflammatory changes in the synovial membrane and cartilage have been reported to develop as early as one day after MIA injection into joints [40]. The degree and rate of articular cartilage degradation and the development of arthrosis are related to the concentration of acid administered to the joint and the duration of its action [41]. As reported by Guingamp et al. [39], high doses of MIA (0.1, 0.3, or 3.0 mg) caused rapid destruction of the treated joint, which involved both cartilage and bone tissue. In addition, on the 1st day after administration of MIA, significant deterioration in the mobility of the joint was caused by pain and inflammation. In turn, low doses (0.01 or 0.03 mg) of MIA did not exert a significant effect on the motor activity of the rats. In our study, we decided to use 0.03 mg of MIA to avoid extensive destruction of the cartilage but better reflect the model of early arthritis. Previous studies showed that this dose initiated early local damage to the hyaline cartilage and caused early degenerative changes [39]. Lower concentrations of MIA were used in the present study. The administration resulted in the development of early degenerative changes in the cartilage and prevented massive joint destruction, which would have significantly limited its functions and impaired the activity of the experimental animals. Similar results were reported by Ferreira-Gomes et al., who used similar concentrations of MIA and detected early degenerative joint disease [42]. The progressive cartilage destruction and development of degenerative joint lesions observed in the analysed animals were accompanied by changes in the bone structure associated with both the MIA administration and the different forms of physical activity [42].

The present study showed a decrease in bone mass density in the immobilised versus active rats, as indicated by the densitometry of the whole body, hind limbs, and knee joints. This confirms a clear impact of physical activity on bone mineralisation. 

In their study, Pereira et al. found that reduced muscle mass and, consequently, muscle strength are responsible for lower bone density and secondary osteoporosis development in elderly females and males [43]. In a study conducted by Verschueren et al., who examined 679 middle-aged and elderly Europeans, subjects with sarcopenia were three times more likely to develop osteoporosis than those with normal muscle mass [44]. Sarcopenia is a term derived from the Greek phrase ‘poverty of flesh’. It was first described in the 1980s as an age-related decline in lean body mass affecting mobility, nutritional status, and independence [45]. Sarcopenia has been defined as a progressive and generalised skeletal muscle disorder involving accelerated loss of muscle mass and function. It is associated with increased adverse outcomes, including falls, functional decline, frailty, and mortality [46]. In the present study, the physically inactive rats were characterised by reduced fat and muscle tissue mass. This may be explained by the phenomenon of the so-called “vicious circle” of musculoskeletal insufficiency in the joint-bone-muscle system. In their study, Fisher et al. observed muscle weakness and a decrease in muscle mass together with the OA progression. This condition was associated with increased pain and progressive disability [47]. Isaac et al. reported that muscle damage was closely related to articular cartilage degeneration [48]. Silva et al. reported a 12–19% decrease in muscle mass in cross sections accompanying degenerative lesions in the hip and knee joints [49]. With the progression of disability and loss of mass and function of periarticular muscles, weakened joint stability and secondary joint hypermobility may be observed. These conditions may accelerate OA development. Hence, the coexistence of OA with sarcopenia is increasingly being reported [50]. As reported by Toda, low muscle quality and loss of lean body mass can be regarded as important risk factors in the pathogenesis of OA [51]. 

Degenerative changes in joints also lead to changes in soft tissues responsible for their motor activity, i.e., atrophy and fatty degeneration of periarticular muscles [52]. Muscle atrophy, which causes functional impairment and disability [53], is usually regarded as a consequence of OA, although the cause-and-effect relationship has not been clearly elucidated [54]. As shown by Rice, muscle weakening is one of the causes of arthrosis development, especially after a joint injury. The injury is followed by neuronal inhibition, which disturbs the proper flow of impulses between the central nervous system and the muscles. This process is known as arthrogenic muscle inhibition (AMI) [55,56]. It is believed that abnormal joint afferent nerves from the osteoarthritic joint lead to neurotransmitter release at the level of the spinal cord, causing a reduction in the activity of alpha motor neurons [57]. As reported by Pap et al., the strength of the quadriceps muscle in knee OA was usually reduced by 14–45%, compared with the control group [58]. In their studies, Nakamura et al. found selective atrophy of type II fibres in OA of the hip and knee joints [59]. The present study demonstrated a higher mass of whole adipose tissue in the hind limbs in the MIA-administered group. This may suggest simultaneous changes in tissues responsible for joint function at each stage of the OA development. This finding was confirmed by Wiewiórski et al., who assessed fatty degeneration of muscles in OA of the ankle joint. The authors found that fatty degeneration mainly affected muscles involved in the motor activity of the OA-affected joint, but the changes involved all hind limb muscles [60].

At the early OA stages, the Hoffa fat pad (IPFP—infrapatellar fat pad) increases its activity. The exact mechanism of the development of these changes in early OA is not clear but may consist in increased production of cytokines, adipokines, interleukins, and growth factors secreted by IPFP, which accelerate cartilage destruction through enhanced production of matrix metalloproteinases [1,61,62]. There are also no data showing changes in the IPFP volume in early OA. The present study showed increased fat tissue mass in the knee joint, which may be related to the Hoffa fat pad mass in this particular measurement. In their study, Hart et al. assessed the volume of the Hoffa fat body in patients after anterior cruciate ligament reconstruction and found that the thickening of the Hoffa fat body may be responsible for the early stage of OA of the entire knee. As suggested by the authors, the volume and metabolic activity of IPFP may be an important marker of early OA [63]. Further studies are required to evaluate IPFP in early OA. On the contrary, at the early stages of OA, Fontanella et al. observed a decrease in the IPFP volume, surface, depth, and femoral and tibial arch lengths as well as an increase in the IPFP hypointense signal in end-stage OA patients compared to meniscal tear and ACLR patients [64].

In conclusion, OA involves changes not only in the articular cartilage but also in the entire musculoskeletal system. The animal model has clearly highlighted the importance of physical activity in the early stages of OA, as it slows down the process of joint destruction as well as bone and muscle atrophy. Physical inactivity in turn contributes to progression of generalised changes in the musculoskeletal system. OA, sarcopenia, osteoporosis, falls, and frailty are not inevitable consequences of aging. In fact, each of these conditions is largely preventable through physical activity [18].

Our study has some possible limitations. The OA model involved the application of a low dose of MIA. However, it will be interesting to compare changes in OA models caused by higher doses. Additionally, there is no comparison between different stages of OA and the duration of the observation. Furthermore, histological examination could be carried out to track the progression of cartilage tissue degradation. On the other hand, the number of manuscripts relating to the early stages of OA and describing the use of Botox to temporary immobilize the limb in an animal model is limited. Further studies will concentrate on middle and late OA stages. Moreover, the different kinds of physical activity could be measured to assess whether different modes and loads of physical activity may influence the result. 

## 4. Materials and Methods

### 4.1. Animal

The study involved 30 male Wistar rats (aged 10–12 weeks) with an average body weight of approx. 210 g. The animals were provided by the company “Hodowla Zwierząt Laboratoryjnych Zbigniew Lipiec ul. Ogrodowa 18, 05-840 Brwinów” (Poland). The study was approved by the 1st Local Ethical Committee for Animal Experiments in Lublin (consent No. 30/2012 of 15 June 2012). The animals were kept in laboratory conditions at a 12:12 light cycle, 21–24 °C temperature, and 30–70% air humidity. The animals had access to fresh water and received a standard rodent diet. The acclimatisation period lasted 7 days.

### 4.2. Experimental Design

After one week (day 0), the animals were allocated to 3 subgroups of 10 animals each. The mean weight of the rats was 210 g and did not differ significantly between the groups. The animals were anaesthetised with the use of a VMS MATRX inhalation anaesthesia machine in a chamber for anaesthesia of laboratory animals and Isoflurane AErrane (BAXTER, Lublin, Poland) at a concentration of 4%. General anaesthesia was maintained with 2% Isoflurane applied through a rodent face mask. 

The animals of each of the three subgroups received monoiodoacetic acid (MIA—Sigma-Aldrich Co., St. Louis, MO, USA) at a concentration of 0.03 mg diluted in 50 μg of sterile saline. It was injected through the patellar ligament of the right knee joint with the use of a U-100 insulin needle. In turn, 50 μg of saline was administered through the patellar ligament in the left knee joint (control).

The rats in the first group were stimulated to exercise on a treadmill (RR—running rats). Three days after the MIA administration, a week-long training period began and the animals ran on a treadmill for 10 min/day at a speed of 10 m/min. After a week, the running time was prolonged to 30 min/day at a speed of 18 m/min. This training regimen was maintained for 3 weeks. The total training time was 28 days.

**Scheme 1:**
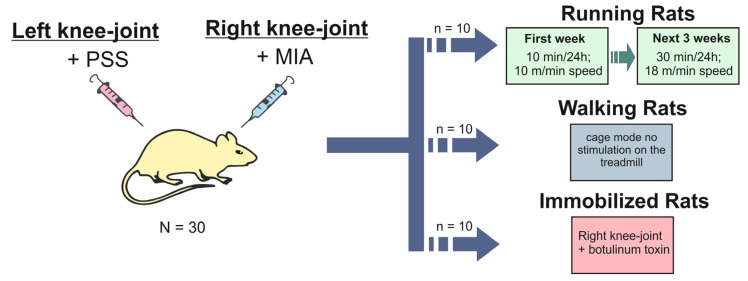
Scheme of experimental procedure. PPS—physiologic saline solution, MIA—monoiodoacetic acid.

The animals in the second group were allowed to lead a natural lifestyle (no treadmill stimulation; WR—walking rats). In the third group, all parts of the right hind limb muscle were injected with Clostridium botulinum toxin type A (botox) supplied by Allergan (Irvine, CA, USA) at a dose of 0.8 units/kg b.w. to disable the movement of the right hind limb (Botox-injected rats, IR—immobilised rats). Experimental design is shown on Scheme 1.

The experiment was terminated on day 28 of the exercise imposed on the RR and WR rats and immobilisation of the IR rats. The animals were anaesthetised via intraperitoneal injection of 10 mg/kg b.w. xylazine (BIOWET, Puławy, Poland) and 80 mg/kg b.w. of ketamine (VET-AGRO, Lublin, Poland). Next, the animals were immobilised on the section table. After exposure of the heart, a 12-gauge needle was inserted into the right ventricle and blood was drained. In the next step, physiological saline was introduced gravitationally into the bloodstream to remove morphotic elements; next, 10% formalin was administered. After ca. 45–60 s, the needle was inserted into the right atrium to fill the vascular bed with formalin.

### 4.3. Radiological Examination 

On the final day of the study, the antero-posterior and lateral x-rays were performed using the Preva Dental X-ray System (Midmark EMEA Ltd., Hampshire, UK). Subsequently, the X-rays were assessed to confirm the early OA stage according to the Kellgren–Lawrence classification. It is a common method of classifying the severity of OA using five grades (Table 4). OA was assessed at the following sites and projections: in the knee it was antero-posterior view.

### 4.4. Body Composition Assessment 

The body composition of the examined animals was assessed using the Lunar Prodigy Advance densitometric apparatus (GE Healthcare, Machelen, Belgium), and the following parameters were determined: body area, BMD—bone mineral density, BMC—bone mineral content expressed in grams (g), total fat mass (Fat mass), and the percentage of fat relative to body weight (Fat%) and muscle mass.

### 4.5. Statistical Analysis and Principle Component Analysis (PCA)

One-way ANOVA followed by Tukey’s post-hoc test (α = 0.05) was used to evaluate the differences between the objects. The calculations were performed using Statstica ver. 13.3 (TIBCO Software Inc., Palo Alto, CA, USA, 2017). In order to reduce the dimensionality of large data sets, the principle component analysis (PCA) was performed based on whole body, knee joint and limb variables (area, BMC, BMD, fat mass, sum of fat-free, and total mass). The coordinates and loadings of each variable were showed in the Table S1.

## Figures and Tables

**Table 1:** Comparison of the parameters of both hind limbs of the animals (right R vs. left L).

	Limb	RR	*p*	WR	*p*	IR	*p*
Area (cm^2^)	R	6.23 ± 0.74	0.9405	6.40 ± 0.41	0.9618	6.10 ± 0.72	0.1259
	L	6.20 ± 0.90		6.42 ± 0.68		6.57 ± 0.78	
BMC (g)	R	0.95 ± 0.08	0.8007	0.97 ± 0.08	0.4211	0.77 ± 0.09	0.0231 *
	L	0.95 ± 0.11		0.95 ± 0.12		0.88 ± 0.12	
BMD (g/cm^2^)	R	0.15 ± 0.02	0.9861	0.15 ± 0.009	0.0534 ^	0.12 ± 0.008	0.0359 *
	L	0.15 ± 0.02		0.14 ± 0.01		0.13 ± 0.007	
Fat mass (g)	R	4.07 ± 1.45	0.4100	3.14 ± 0.72	0.1376	4.16 ± 1.12	0.3384
	L	3.88 ± 0.97		2.67 ± 0.74		4.52 ± 1.6	
Fat-free mass (g)	R	23.7 ± 2.68	0.2525	23.76 ± 2.84	0.1036	20.24 ± 3.33	0.0120 *
	L	22.75 ± 2.27		21.77 ± 2.15		23.62 ± 4.54	
Mass (g)	R	28.19 ± 4.93	0.3600	26.86 ± 3.04	0.0960	24.42 ± 4.08	0.0200 *
	L	26.94 ± 2.59		23.87 ± 3.28		28.12 ± 6.01	
Fat (%)	R	14.15 ± 3.25	0.4900	11.62 ± 2.07	0.3740	16.91 ± 3.04	0.1418
	L	14.37 ± 3.43		11.02 ± 1.78		15.65 ± 2.8	

BMC—bone mineral content, BMD—bone mineral density, RR—running rats, WR—walking rats, IR—immobilised rats, * means statistically significant difference, ^—means value close to statistical significance difference (α = 0.05).

**Table 2:** Comparison of the parameters of both knee joints of the animals (right R vs. left L).

	Knee Joint	RR	*p*	WR	*p*	IR	*p*
Area (cm^2^)	R	2.18 ± 0.18	0.4443	2.12 ± 0.24	0.125	1.90 ± 0.15	0.0073 *
	L	2.26 ± 0.28		2.31 ± 0.35		2.2 ± 0.24	
BMC (g)	R	0.37 ± 0.07	0.7950	0.32 ± 0.04	0.5062	0.2 ± 0.02	0.0001 *
	L	0.39 ± 0.13		0.33 ± 0.05		0.28 ± 0.04	
BMD (g/cm^2^)	R	0.17 ± 0.03	0.9679	0.15 ± 0.01	0.3992	0.11 ± 0.01	0.0008 *
	L	0.17 ± 0.05		0.15 ± 0.01		0.13 ± 0.01	
Fat mass (g)	R	1.11 ± 0.38	0.7912	0.65 ± 0.22	0.0462 *	0.95 ± 0.29	0.0011 *
	L	1.23 ± 1.04		0.48 ± 0.09		0.6 ± 0.2	
Fat-free mass (g)	R	7.07 ± 2.49	0.9950	5.75 ± 1.33	0.2948	4.62 ± 0.6	0.3397
	L	7.37 ± 3.86		5.24 ± 1.08		4.28 ± 0.58	
Mass (g)	R	8.25 ± 2.58	0.700	6.56 ± 1.41	0.1100	5.48 ± 0.54	0.2400
	L	8.31 ± 4.62		5.8 ± 1.11		5.01 ± 0.73	
Fat (%)	R	13.6 ± 3.72	0.4100	10.19 ± 2.88	0.0430 *	17.15 ± 5.00	0.002 *
	L	12.87 ± 3.51		8.39 ± 1.51		10.95 ± 2.47	

BMC—bone mineral content, BMD—bone mineral density, RR—running rats, WR—walking rats, IR—immobilised rats, * means statistically significant difference (α = 0.05).

**Table 3:** Right limb and right knee variables. Data are means ± SD (*n* = 8–9).

		RR	WR	IR	*p* WR vs. RR	*p* RR vs. IR	*p* WR vs. IR
Area (cm^2^)	Limb	6.23 ± 0.74	6.40 ± 0.41	6.10 ± 0.72	0.3900	0.5300	0.5200
	Knee joint	2.18 ± 0.18	2.12 ± 0.24	1.90 ± 0.15	0.3103	0.0010 *	0.0990
BMC (g)	Limb	0.95 ± 0.08	0.97 ± 0.08	0.77 ± 0.09	0.7041	0.0006 *	0.0087 *
	Knee joint	0.37 ± 0.07	0.32 ± 0.04	0.20 ± 0.02	0.2573	0.0006 *	0.0013 *
BMD (g/cm^2^)	Limb	0.15 ± 0.02	0.15 ± 0.009	0.12 ± 0.008	0.6048	0.0131 *	0.0005 *
	Knee joint	0.17 ± 0.03	0.15 ± 0.01	0.11 ± 0.01	0.3849	0.0079 *	0.0003 *
Fat mass (g)	Limb	4.07 ± 1.45	3.14 ± 0.72	4.16 ± 1.12	0.1200	0.8225	0.0093 *
	Knee joint	1.11 ± 0.38	0.65 ± 0.22	0.95 ± 0.29	0.0284 *	0.7970	0.0228 *
Fat-free mass (g)	Limb	23.70 ± 2.68	23.76 ± 2.84	20.24 ± 3.33	0.9339	0.1591	0.1385
	Knee joint	7.07 ± 2.49	5.75 ± 1.33	4.62 ± 0.6	0.3858	0.0202 *	0.0608 ^
Mass (g)	Limb	28.19 ± 4.93	26.86 ± 3.04	24.42 ± 4.08	0.4000	0.2500	0.5800
	Knee joint	8.25 ± 2.58	6.56 ± 1.41	5.48 ± 0.54	0.2800	0.0080 *	0.0700 ^
Fat (%)	Limb	14.15 ± 3.25	11.62 ± 2.07	16.91 ± 3.04	0.0875	0.0972	0.0004 *
	Knee joint	13.6 ± 3.72	10.19 ± 2.88	17.15 ± 5.00	0.2849	0.0234 *	0.1531

BMC—bone mineral content, BMD—bone mineral density, RR—running rats, WR—walking rats, IR—immobilised rats, * means statistically significant difference, ^—means value close to statistical significance difference (α = 0.05).

**Table 4:** Kellgren–Lawrence classification.

Grade	Severity of OA
Grade 0	definite absence of X-ray changes of osteoarthritis
Grade I	doubtful joint space narrowing and possible osteophytic lipping
Grade II	definite osteophytes and possible joint space narrowing
Grade III	moderate multiple osteophytes, definite narrowing of joint space and some sclerosis and possible deformity of bone ends
Grade IV	large osteophytes, marked narrowing of joint space, severe sclerosis and definite deformity of bone ends

## Data Availability

The data presented in this study are available on request from the corresponding author.

## Supplementary Materials

The following supporting information can be downloaded at: https://www.mdpi.com/article/10.3390/ijms24043540/s1.

## References

[1] Jarecki J., Małecka-Massalska T., Polkowska I., Potoczniak B., Kosior-Jarecka E., Szerb I., Tomaszewska E., Gutbier M., Dobrzyński M., Blicharski T. (2021). Level of Adiponectin, Leptin and Selected Matrix Metalloproteinases in Female Overweight Patients with Primary Gonarthrosis. J. Clin. Med..

[2] Karpiński R. (2022). Knee joint osteoarthritis diagnosis based on selected acoustic signal discriminants using machine learning. Appl. Comput. Sci..

[3] Krakowski P., Karpiński R., Maciejewski R., Jonak J., Jurkiewicz A. (2020). Short-Term Effects of Arthroscopic Microfracturation of Knee Chondral Defects in Osteoarthritis. Appl. Sci..

[4] Englund M., Guermazi A., Lohmander S.L. (2009). The Role of the Meniscus in Knee Osteoarthritis: A Cause or Consequence?. Radiol. Clin. N. Am..

[5] Donell S. (2019). Subchondral Bone Remodelling in Osteoarthritis. EFORT Open Rev..

[6] Belluzzi E., Macchi V., Fontanella C., Carniel E., Olivotto E., Filardo G., Sarasin G., Porzionato A., Granzotto M., Pozzuoli A. (2020). Infrapatellar Fat Pad Gene Expression and Protein Production in Patients with and without Osteoarthritis. Int. J. Mol. Sci..

[7] Hunter D.J., Bierma-Zeinstra S. (2019). Osteoarthritis. Lancet.

[8] Dainese P., Wyngaert K.V., De Mits S., Wittoek R., Van Ginckel A., Calders P. (2022). Association between Knee Inflammation and Knee Pain in Patients with Knee Osteoarthritis: A Systematic Review. Osteoarthr. Cartil..

[9] Kirwan R., McCullough D., Butler T., Perez de Heredia F., Davies I.G., Stewart C. (2020). Sarcopenia during COVID-19 Lockdown Restrictions: Long-Term Health Effects of Short-Term Muscle Loss. GeroScience.

[10] Martin A.C. (2011). Osteoporosis in Men: A Review of Endogenous Sex Hormones and Testosterone Replacement Therapy. J. Pharm. Pr..

[11] Halloran B.P., Bikle D.D., Harris J., Autry C.P., Currier P.A., Tanner S., Patterson-Buckendahl P., Morey-Holton E. (1995). Skeletal Unloading Induces Selective Resistance to the Anabolic Actions of Growth Hormone on Bone. J. Bone Miner. Res..

[12] McLean R.R. (2009). Proinflammatory Cytokines and Osteoporosis. Curr. Osteoporos. Rep..

[13] Cederholm T., Cruz-Jentoft A.J., Maggi S. (2013). Sarcopenia and Fragility Fractures. Eur. J. Phys. Rehabil. Med..

[14] Siddiq A., Ansari M.O., Mohammad A., Mohammad F., El-Desoky G.E. (2014). Synergistic Effect of Polyaniline Modified Silica Gel for Highly Efficient Separation of Non Resolvable Amino Acids. Int. J. Polym. Mater. Polym. Biomater..

[15] Jarecki J., Sobiech M., Turżańska K., Tomczyk-Warunek A., Jabłoński M. (2021). A Kinesio Taping Method Applied in the Treatment of Postsurgical Knee Swelling after Primary Total Knee Arthroplasty. J. Clin. Med..

[16] Krakowski P., Karpiński R., Jojczuk M., Nogalska A., Jonak J. (2021). Knee MRI Underestimates the Grade of Cartilage Lesions. Appl. Sci..

[17] L’Hermette M.F., Tourny-Chollet C., Polle G., Dujardin F.H. (2006). Articular Cartilage, Degenerative Process, and Repair: Current Progress. Int. J. Sports Med..

[18] Eckstrom E., Neukam S., Kalin L., Wright J. (2020). Physical Activity and Healthy Aging. Clin. Geriatr. Med..

[19] Messina O.D., Vidal Wilman M., Vidal Neira L.F. (2019). Nutrition, Osteoarthritis and Cartilage Metabolism. Aging Clin. Exp. Res..

[20] Daste C., Kirren Q., Akoum J., Lefèvre-Colau M.-M., Rannou F., Nguyen C. (2021). Physical Activity for Osteoarthritis: Efficiency and Review of Recommandations. Jt. Bone Spine.

[21] Berenbaum F., Wallace I.J., Lieberman D.E., Felson D.T. (2018). Modern-Day Environmental Factors in the Pathogenesis of Osteoarthritis. Nat. Rev. Rheumatol..

[22] Rosenberg I.H. (1989). Summary Comments. Am. J. Clin. Nutr..

[23] Santilli V., Bernetti A., Mangone M., Paoloni M. (2014). Clinical Definition of Sarcopenia. Clin. Cases Miner Bone. Metab..

[24] Cruz-Jentoft A.J., Sayer A.A. (2019). Sarcopenia. Lancet.

[25] Cruz-Jentoft A.J. (2016). Sarcopenia, the Last Organ Insufficiency. Eur. Geriatr. Med..

[26] Papalia R., Zampogna B., Torre G., Lanotte A., Vasta S., Albo E., Tecame A., Denaro V. (2014). Sarcopenia and Its Relationship with Osteoarthritis: Risk Factor or Direct Consequence?. Musculoskelet. Surg..

[27] Li C., Yu K., Shyh-Chang N., Li G., Jiang L., Yu S., Xu L., Liu R., Guo Z., Xie H. (2019). Circulating Factors Associated with Sarcopenia during Ageing and after Intensive Lifestyle Intervention. J. Cachexia Sarcopenia Muscle.

[28] Scott D., Blizzard L., Fell J., Jones G. (2012). Prospective Study of Self-Reported Pain, Radiographic Osteoarthritis, Sarcopenia Progression, and Falls Risk in Community-Dwelling Older Adults. Arthritis Care Res..

[29] Grad S., Eglin D., Alini M., Stoddart M.J. (2011). Physical Stimulation of Chondrogenic Cells In Vitro: A Review. Clin. Orthop. Relat. Res..

[30] Rannou F., Lee T.-S., Zhou R.-H., Chin J., Lotz J.C., Mayoux-Benhamou M.-A., Barbet J.P., Chevrot A., Shyy J.Y.-J. (2004). Intervertebral Disc Degeneration. Am. J. Pathol..

[31] Lee S., Kim T.-N., Kim S.-H. (2012). Sarcopenic Obesity Is More Closely Associated with Knee Osteoarthritis than Is Nonsarcopenic Obesity: A Cross-Sectional Study. Arthritis Rheum..

[32] Lee S.Y., Ro H.J., Chung S.G., Kang S.H., Seo K.M., Kim D.-K. (2016). Low Skeletal Muscle Mass in the Lower Limbs Is Independently Associated to Knee Osteoarthritis. PLoS ONE.

[33] Toda Y., Segal N., Toda T., Kato A., Toda F. (2000). A Decline in Lower Extremity Lean Body Mass per Body Weight Is Characteristic of Women with Early Phase Osteoarthritis of the Knee. J. Rheumatol..

[34] Veronese N., Stefanac S., Koyanagi A., Al-Daghri N.M., Sabico S., Cooper C., Rizzoli R., Reginster J.-Y., Barbagallo M., Dominguez L.J. (2021). Lower Limb Muscle Strength and Muscle Mass Are Associated with Incident Symptomatic Knee Osteoarthritis: A Longitudinal Cohort Study. Front. Endocrinol..

[35] Peng H., Zeng Y. (2022). Research progress on the correlation between sarcopenia and osteoarthritis. Zhongguo Xiu Fu Chong Jian Wai Ke Za Zhi.

[36] Kellgren J.H., Lawrence J.S. (1957). Radiological Assessment of Osteo-Arthrosis. Ann. Rheum. Dis..

[37] Karpiński R., Krakowski P., Jonak J., Machrowska A., Maciejewski M., Nogalski A. (2022). Diagnostics of Articular Cartilage Damage Based on Generated Acoustic Signals Using ANN—Part I: Femoral-Tibial Joint. Sensors.

[38] Bove S.E., Calcaterra S.L., Brooker R.M., Huber C.M., Guzman R.E., Juneau P.L., Schrier D.J., Kilgore K.S. (2003). Weight Bearing as a Measure of Disease Progression and Efficacy of Anti-Inflammatory Compounds in a Model of Monosodium Iodoacetate-Induced Osteoarthritis. Osteoarthr. Cartil..

[39] Guingamp C., Gegout-Pottie P., Philippe L., Terlain B., Netter P., Gillet P. (1997). Mono-Iodoacetate-Induced Experimental Osteoarthritis. A Dose-Response Study of Loss of Mobility, Morphology, and Biochemistry. Arthritis Rheum..

[40] Orita S., Ishikawa T., Miyagi M., Ochiai N., Inoue G., Eguchi Y., Kamoda H., Arai G., Toyone T., Aoki Y. (2011). Pain-Related Sensory Innervation in Monoiodoacetate-Induced Osteoarthritis in Rat Knees That Gradually Develops Neuronal Injury in Addition to Inflammatory Pain. BMC Musculoskelet. Disord..

[41] Kobayashi K., Imaizumi R., Sumichika H., Tanaka H., Goda M., Fukunari A., Komatsu H. (2003). Sodium Iodoacetate-Induced Experimental Osteoarthritis and Associated Pain Model in Rats. J. Vet. Med. Sci..

[42] Ferreira-Gomes J., Adães S., Sousa R.M., Mendonça M., Castro-Lopes J.M. (2012). Dose-Dependent Expression of Neuronal Injury Markers during Experimental Osteoarthritis Induced by Monoiodoacetate in the Rat. Mol. Pain.

[43] Pereira F.B., Leite A.F., de Paula A.P. (2015). Relationship between Pre-Sarcopenia, Sarcopenia and Bone Mineral Density in Elderly Men. Arch. Endocrinol. Metab..

[44] Verschueren S., Gielen E., O’Neill T.W., Pye S.R., Adams J.E., Ward K.A., Wu F.C., Szulc P., Laurent M., Claessens F. (2013). Sarcopenia and Its Relationship with Bone Mineral Density in Middle-Aged and Elderly European Men. Osteoporos. Int..

[45] Rosenberg I.H. (1997). Sarcopenia: Origins and Clinical Relevance. J. Nutr..

[46] Cruz-Jentoft A.J., Bahat G., Bauer J., Boirie Y., Bruyère O., Cederholm T., Cooper C., Landi F., Rolland Y., Sayer A.A. (2019). Sarcopenia: Revised European Consensus on Definition and Diagnosis. Age Ageing.

[47] Fisher N.M., White S.C., Yack H.J., Smolinski R.J., Pendergast D.R. (1997). Muscle Function and Gait in Patients with Knee Osteoarthritis before and after Muscle Rehabilitation. Disabil. Rehabil..

[48] Isaac C., Wright A., Usas A., Li H., Tang Y., Mu X., Greco N., Dong Q., Vo N., Kang J. (2013). Dystrophin and Utrophin “Double Knockout” Dystrophic Mice Exhibit a Spectrum of Degenerative Musculoskeletal Abnormalities. J. Orthop. Res..

[49] de Silva J.M.S., Alabarse P.V.G., Teixeira V.d.O.N., Freitas E.C., de Oliveira F.H., Chakr R.M.d.S., Xavier R.M. (2018). Muscle Wasting in Osteoarthritis Model Induced by Anterior Cruciate Ligament Transection. PLoS ONE.

[50] Larsson L., Degens H., Li M., Salviati L., Lee Y., Thompson W., Kirkland J.L., Sandri M. (2019). Sarcopenia: Aging-Related Loss of Muscle Mass and Function. Physiol. Rev..

[51] Toda Y., Kobayashi T. (2000). The Usefulness of Walking for Preventing Sarcopenia in Dieting Postmenopausal Women Complaining of Knee Pain. Ann. N. Y. Acad. Sci..

[52] Sίrca A., Susěc-Michieli M. (1980). Selective Type II Fibre Muscular Atrophy in Patients with Osteoarthritis of the Hip. J. Neurol. Sci..

[53] Janssen I., Heymsfield S.B., Ross R. (2002). Low Relative Skeletal Muscle Mass (Sarcopenia) in Older Persons Is Associated with Functional Impairment and Physical Disability. J. Am. Geriatr. Soc..

[54] Hurley M.V. (1999). The Role of Muscle Weakness in the Pathogenesis of Osteoarthritis. Rheum. Dis. Clin. N. Am..

[55] Rice D.A., McNair P.J. (2010). Quadriceps Arthrogenic Muscle Inhibition: Neural Mechanisms and Treatment Perspectives. Semin. Arthritis Rheum..

[56] Zachařová G., Knotková-Urbancová H., Hník P., Soukup T. (1997). Nociceptive Atrophy of the Rat Soleus Muscle Induced by Bone Fracture: A Morphometric Study. J. Appl. Physiol..

[57] Hurley M.V., Scott D.L., Rees J., Newham D.J. (1997). Sensorimotor Changes and Functional Performance in Patients with Knee Osteoarthritis. Ann. Rheum. Dis..

[58] Pap G., Machner A., Awiszus F. (2004). Strength and Voluntary Activation of the Quadriceps Femoris Muscle at Different Severities of Osteoarthritic Knee Joint Damage. J. Orthop. Res..

[59] Nakamura T., Suzuki K. (1992). Muscular Changes in Osteoarthritis of the Hip and Knee. Nihon Seikeigeka Gakkai Zasshi.

[60] Wiewiorski M., Dopke K., Steiger C., Valderrabano V. (2012). Muscular Atrophy of the Lower Leg in Unilateral Post Traumatic Osteoarthritis of the Ankle Joint. Int. Orthop. (SICOT).

[61] Braun S., Zaucke F., Brenneis M., Rapp A.E., Pollinger P., Sohn R., Jenei-Lanzl Z., Meurer A. (2022). The Corpus Adiposum Infrapatellare (Hoffa’s Fat Pad)—The Role of the Infrapatellar Fat Pad in Osteoarthritis Pathogenesis. Biomedicines.

[62] Jarecki J., Małecka-Masalska T., Kosior-Jarecka E., Widuchowski W., Krasowski P., Gutbier M., Dobrzyński M., Blicharski T. (2022). Concentration of Selected Metalloproteinases and Osteocalcin in the Serum and Synovial Fluid of Obese Women with Advanced Knee Osteoarthritis. Int. J. Environ. Res. Public Health.

[63] Hart H.F., Culvenor A.G., Patterson B.E., Doshi A., Vora A., Guermazi A., Birmingham T.B., Crossley K.M. (2022). Infrapatellar Fat Pad Volume and Hoffa-synovitis after ACL Reconstruction: Association with Early Osteoarthritis Features and Pain over 5 Years. J. Orthop. Res..

[64] Fontanella C.G., Belluzzi E., Pozzuoli A., Scioni M., Olivotto E., Reale D., Ruggieri P., De Caro R., Ramonda R., Carniel E.L. (2022). Exploring Anatomo-Morphometric Characteristics of Infrapatellar, Suprapatellar Fat Pad, and Knee Ligaments in Osteoarthritis Compared to Post-Traumatic Lesions. Biomedicines.

