# Supplementary material for: Assessment of the Impact of Physical Activity on the Musculoskeletal System in Early Degenerative Knee Joint Lesions in an Animal Model
Source: Int J Mol Sci. 2023 Feb 10;24(4):3540. doi: 10.3390/ijms24043540 (PMC9960538; doi:10.3390/ijms24043540)
Supplement: Supplementary file 1 [file ijms-24-03540-s001.zip › ijms-2189817-supplementary.pdf]

International Journal of *Molecular Sciences*

MDPI

Supplementary material

Assessment of the impact of physical activity on the musculoskeletal system in early degenerative knee joint lesions in an animal model

Jaromir Jarecki[1]*, Izabela Polkowska[2], Waldemar Kazimierczak[3], Magdalena Wójciak[4], Ireneusz Sowa[4] and Sławomir Dresler [4,5]

[1] Department of Rehabilitation and Orthopaedics, Medical University of Lublin, 20-059 Lublin, Poland
[2] Department and Clinic of Animal Surgery, University of Life Sciences, 20-033 Lublin, Poland
[3] Department of Biomedicine and Environmental Research Faculty of Medicine John Paul II Catholic University of Lublin; 20-708 Lublin
[4] Department of Analytical Chemistry, Medical University of Lublin, Aleje Raclawickie 1, 20-059 Lublin, Poland;
[5] Department of Plant Physiology and Biophysics, Institute of Biological Sciences, Maria Curie-Skłodowska University, 20-033 Lublin, Poland
* Correspondence: jaromirj@interia.pl

Table S1. Factor loadings between variables and principal components.

**Variables**	**PC 1**	**PC 2**
Body area	-0.682	-0.115
BMC	-0.830	-0.367
BMD	-0.648	-0.471
Fat mass	-0.827	0.312
Sum of fat-free and total mass	-0.826	-0.317
Total mass	-0.892	-0.167
Fat	-0.603	0.558
Limb area (R)	-0.262	0.334
Limb area (L)	-0.207	0.068
Limb BMC (R)	-0.591	-0.511
Limb BMC (L)	-0.772	-0.092
Limb BMD (R)	-0.413	-0.785
Limb BMD (L)	-0.712	-0.132
Limb fat mass (R)	-0.685	0.616
Limb fat mass (L)	-0.523	0.653
Sum of fat-free and total mass (Limb R)	-0.599	-0.176
Sum of fat-free and total mass (Limb L)	-0.357	0.185
Limb mass (R)	-0.697	0.013
Limb mass (L)	-0.433	0.331

Percent of fat (Limb R)	-0.345	0.804
Percent of fat (Limb L)	-0.503	0.718
Knee-joint area (R)	-0.712	-0.250
Knee-joint area (L)	-0.428	0.091
Knee-joint BMC (R)	-0.552	-0.763
Knee-joint BMC (L)	-0.658	0.015
Knee-joint BMD (R)	-0.386	-0.815
Knee-joint BMD (L)	-0.565	-0.037
Knee-joint fat mass (R)	-0.494	0.240
Knee-joint fat mass (L)	-0.671	0.338
Sum of fat-free and total mass (Knee-joint R)	-0.382	-0.644
Sum of fat-free and total mass (Knee-joint L)	-0.560	0.103
Knee-joint mass (R)	-0.438	-0.551
Knee-joint mass (L)	-0.593	0.151
Percent of fat (Knee-joint R)	-0.233	0.774
Percent of fat (Knee-joint L)	-0.609	0.623

R- right side, L- left side, BMC- bone mineral content, BMD-bone mineral density, PC- principal component.
